# Directly Writing Resistor, Inductor and Capacitor to Composite Functional Circuits: A Super-Simple Way for Alternative Electronics

**DOI:** 10.1371/journal.pone.0069761

**Published:** 2013-08-02

**Authors:** Yunxia Gao, Haiyan Li, Jing Liu

**Affiliations:** 1 Key Lab of Cryogenics and Beijing Key Lab of CryoBiomedical Engineering, Technical Institute of Physics and Chemistry, Chinese Academy of Sciences, Beijing, China; 2 Department of Biomedical Engineering, School of Medicine, Tsinghua University, Beijing, China; Texas A&M University, United States of America

## Abstract

**Background:**

The current strategies for making electronic devices are generally time, water, material and energy consuming. Here, the direct writing of composite functional circuits through comprehensive use of GaIn_10_-based liquid metal inks and matching material is proposed and investigated, which is a rather easy going and cost effective electronics fabrication way compared with the conventional approaches.

**Methods:**

Owing to its excellent adhesion and electrical properties, the liquid metal ink was demonstrated as a generalist in directly making various basic electronic components such as planar resistor, inductor and capacitor or their combination and thus composing circuits with expected electrical functions. For a precise control of the geometric sizes of the writing, a mask with a designed pattern was employed and demonstrated. Mechanisms for justifying the chemical components of the inks and the magnitudes of the target electronic elements so as to compose various practical circuits were disclosed.

**Results:**

Fundamental tests on the electrical components including capacitor and inductor directly written on paper with working time up to 48 h and elevated temperature demonstrated their good stability and potential widespread adaptability especially when used in some high frequency circuits. As the first proof-of-concept experiment, a typical functional oscillating circuit including an integrated chip of 74HC04 with a supply voltage of 5 V, a capacitor of 10 nF and two resistors of 5 kΩ and 1 kΩ respectively was directly composed on paper through integrating specific electrical elements together, which presented an oscillation frequency of 8.8 kHz.

**Conclusions:**

The present method significantly extends the roles of the metal ink in recent works serving as only a single electrical conductor or interconnecting wires. It opens the way for directly writing out complex functional circuits or devices on different substrates. Such circuit composition strategy has generalized purpose and can be extended to more areas, even daily pervasive electronics.

## Introduction

Recently, there is an increasing demand in electronics area to develop innovative fabrication strategies that are efficient, time, water, material and energy saving [1], [2]. Among the many efforts ever made, the classical technologies such as lithography, imprinting etc. have significantly stimulated the rapid progress of the science and technology. An undesirable feature for these methods is that they are generally complex, expensive and thus not easily available. As an alternative, the newly emerging direct writing technology, which possesses a sophisticated and refined patterning ability, opens promising future [3], especially in those areas like flexible displays [4]–[6], conformal antenna arrays, thin film transistors [7]–[12], membrane keyboards, electronic solar cell arrays [13], [14], radio-frequency identification (RFID) tags [15], flexible batteries [16], [17], and electronic circuits fabricated in e.g. clothing or biomedical devices etc. [18]–[21]. Direct writing therefore appeared as a rather effective philosophy in the electronic fabrication due to their huge potential values [3]. The core of such practices lies in the development of electronic-inks with various superior physical or chemical properties.

It is well known that conventional functional inks generally include nano-sized metal powders, high molecular weight polymers, and ceramic materials which are important and integral parts of the direct writing practices. Although considerable efforts have been made on developing conductive inks for the direct writing technology, many of them are found to be not idealistic enough. Taking the conductive inks for instance, one of the major issues is that most of the existing inks still have a pretty low conductivity. Poly(3,4-ethylenedioxythiophene) doped with poly(4-styrenesulfonate) (PEDOT:PSS), one of the organic conductive materials with good properties, has attracted much attention and is being used as electrodes for capacitors or photodiodes. Many works have reported on the conductivity modification of PEDOT: PSS by adding several additives such as polyols, metals and different solvents [22], [23]. In such endeavor, a very high heat treatment temperature has to be adopted to improve the conductivity of the ink. It is shown that the synthesized inks could retain the conductivity of about 50% of the bulk after heat treatment at around 400°C when their wet patterns are formulated [3]. Unfortunately, vast amounts of plastic substrates require the post heat treatment below 150°C and under such a situation, the conductivity of the final patterns would exhibit only 30% of the bulk metals [3]. Meanwhile, researches also turn to paper substrates because it can be used as a low-cost, enabling platform for many flexible and disposable devices [24], [25]. In fact, paper substrate offers tremendous advantages for printed electronic devices. It is not only widely available and inexpensive, but also lightweight, biodegradable, and can be rolled or folded into 3D configurations. However the heat treatment temperature of paper must drop below 100°C, which may result in a much lower conductivity of conventional inks.

Overall, for the existing direct writing methods, there still inherit a series of undesirable features such as higher cost, easy oxidation, lower electrical resistivity and adhesiveness, and complicated preparation process. Clearly, the success of the direct writing technology urgently requests explosive innovation which should be fundamentally different in a large extent from the currently available technical routes.

Starting from this basic consideration, we recently turn our vision to the room temperature liquid metal, whose role as electronic ink was unfortunately neglected before. As has been clarified, gallium and its alloys all have very low vapor pressure. According to some studies [26], [27], Galinstan, one of the gallium alloys, has been used successfully as a dental filling alloy. Compared with the conventional silver chemicals, such materials own many unique favorable virtues such as low enough melting point around room temperature, extremely simple preparation process and so on [28]. For example, the silver ink is prepared by silver particles in an aqueous solution by reducing silver nitrate in the presence of a surface capping agent, poly (acrylic acid) and diethanolamine [29]–[31], while liquid metal ink can be fabricated by a rather straightforward oxidation reaction within only several minutes. And the liquid metal ink based conductive film does not require any heat treatments for improving its electrical conductivity any more. In addition, it still owns some other advantages. For example, the used liquid metal ink can be reclaimed efficiently by 30% NaOH solution. The reclamation and recycle of liquid metal ink not only can control pollution but also solve the problems of waste of raw materials. Besides, the semi-liquid state of the liquid metal ink is helpful for self-healing when the conductive pathway is broken. Blaiszik *et al* proposed a self-healing circuit and the liquid GaIn alloy encapsulated in a polymeric urea-formaldehyde shell wall is patterned on the conductive lines to heal with the broken circuits timely [32]. The autonomic restoration of electrical conductivity in a damaged circuit would lead to increased longevity and device reliability and effectively raise the working efficiency.

As a very initial attempt, we have tried before to use the metal ink to directly write out electrical interconnects, which clearly showed promising features of the new technique [33]. To further significantly extend the increasing use of the liquid metal based printed electronics, we are dedicated here to explore its capacity in directly writing out a complete functional circuit. To illustrate the basic principle of the current strategy, three most fundamental elements in an electrical circuit including resistor, inductor and capacitor and their combination are written on paper with liquid metal ink for the first time, which paved the way for constructing various complex electronics and circuits even devices. The magnitudes of the fabricated electronic elements are found to be easily controllable through geometrical or chemical components design. The adoption of the present method in electronics area opens the way for quickly writing out the whole circuit components with desired functions which can be as simple as drawing a picture on the paper. Such electronics making strategy has generalized purpose and can be extended to more areas, even daily life.

## Materials and Methods

### Preparation of GaIn_10_-Based Liquid Metal Ink

Gallium and indium metals with purity of 99.99 percent used as raw materials were weighted with a weight ratio of 90∶10 according to the chemical composition of GaIn_10_ alloy. The detailed synthesis procedures were outlined respectively as follows [33]: First, the gallium was added into the beaker and reduced to liquid form through heating at 373 K. Then indium with a melting point of 429.8 K was loaded into the liquid gallium to prepare GaIn_10_ alloy. The mixtures were stirred using a magnetic stirrer when they were all melted. Then 10 ml 30% NaOH solution was added slowly to clean the metals. The mixture was stirred at room temperature for a short period of time. The contents of the beaker had both an aqueous phase and a metallic phase. Then, the GaIn_10_ alloy was separated from the mixture and stirred constantly in air at room temperature to be oxidized. The oxide layer formed easily on the surface of alloys was broken by the vigorous stirring so that more and more gallium metal can be oxidized. With the increase of the formed gallium oxides, the viscosity of the alloy can be improved. After being vigorously stirred for 10 min with a stir speed of 200 r min^−1^, one can get the GaIn_10_-based liquid metal inks with an appropriate viscosity, which was composed of GaIn_10_ alloy and a little amount of gallium oxide dispersed uniformly in alloy. It was worth to mention that in this experiment, several parameters such as amount of gallium, stir time and stir speed should be tightly controlled.

### Characterization of GaIn_10_-Based Liquid Metal Ink

The melting point of gallium-based liquid metal ink was measured by the differential scanning calorimeter (DSC 200 F3 Maia, Netzsch). The surface morphology and thickness of the conductive line written on papers were observed with field-emission scanning electron microscope (FESEM, S4300, Hitachi). Electrical resistivity measurements were performed using four point probe method in air at room temperature which were measured with an accuracy ±0.5°C by T-type thermocouples. The data were recorded using Agilent 34972A (USA) which was a data logger to acquire voltage, current and temperature signals. Prior to each measurement, the sample was stabilized at constant temperature for at least 2 h to ensure thermal equilibrium. Contact angles were measured by the contact angle instrument (JC2000D3) at room temperature. The measurements of capacitance, inductance and resistance were performed using a LCR digital electric bridge. The measurement frequency and the level were chosen to be 100 kHz and 0.3 V, respectively.

## Experimental Results

### Direct Writing of Fundamental Electronic Components

Although there are quite a few candidates for room temperature liquid metal inks which guarantee the wide acceptability of the new method, here only the gallium based alloy is investigated for brevity. GaIn_10_ liquid metal ink (weight percent, Ga90%, In10%) is prepared by controlling an oxidization reaction. The content of gallium oxides has been controlled by adjusting the experimental parameters. In the present work, the GaIn_10_-based liquid metal ink consists of 0.026 wt% oxygen which exists in the form of gallium oxides and can be calculated by the weight increase of GaIn_10_-based liquid metal with and without oxidization, respectively.

As our recent experiments disclose, the existence of gallium oxides significantly improves the adhesion of GaIn_10_-based liquid metal with different substrates [28], [33]. GaIn_10_-based ink consists of 0.026 wt% oxygen has been measured as 34.5 µΩ·cm at 297 K by four point probe method [33]. Now, it was clear that the GaIn_10_-based liquid metal ink with only 0.026% oxygen prepared by oxidation reaction method can be directly and easily written on paper using different kinds of handy tools such as ball-pen, brush pen and so on. The only difference between the present approach and the conventional writing or drawing in daily life is that the liquid metal conductive ink is used here to substitute the water like ink. In this way, people can write out various shapes and patterns conveniently on the flexible substrates according to specific demands of the target electrical devices owing to the excellent wettability of the liquid metal ink with other materials.

**Figure 1:**
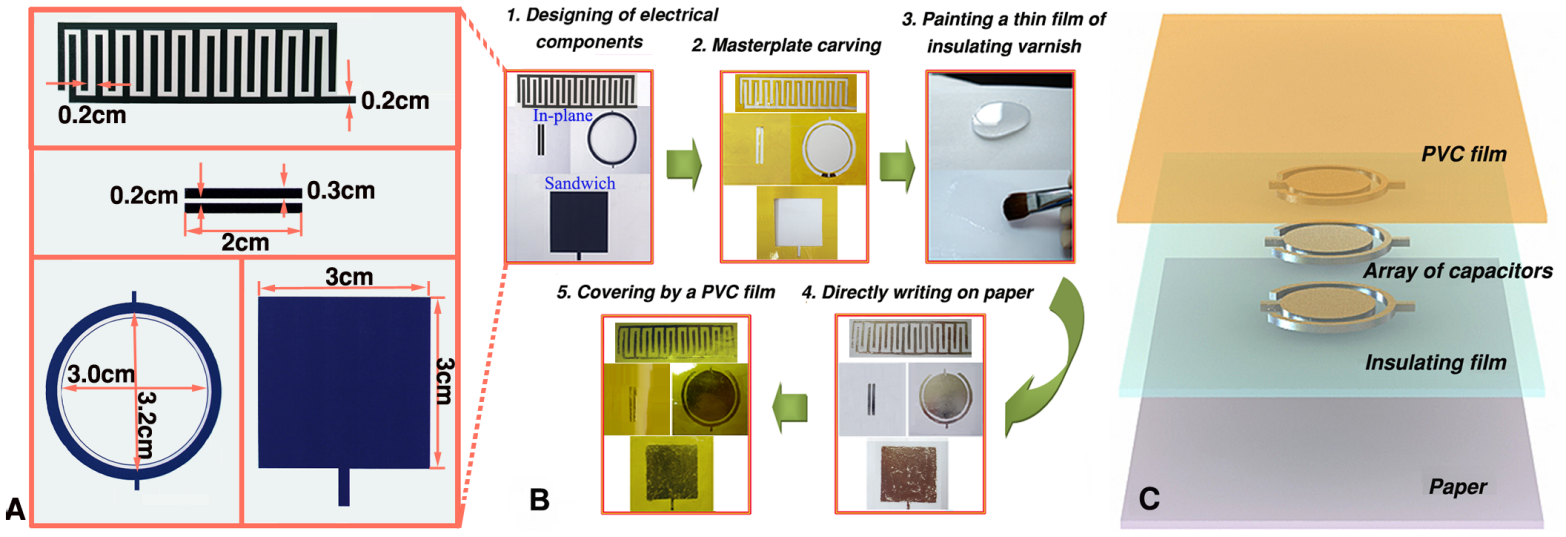
The direct writing process of electrical components on paper by using GaIn_10_-based liquid metal ink. (A) The detailed dimensions of both in-plane and conventional sandwich patterns of the designed capacitors. (B) The preparation process of both in-plane and conventional sandwich patterns of electrical capacitors are taken for an example. (C) Exploded view schematic illustration of an array of concentric circular patterns of electrical capacitors written in the same plane.

As is well known, resistor, capacitor and inductor are three most basic electrical components which are widely used in electrical circuits design, such as various LC\RC\LCR devices and some other complicated functional circuits. Here, the capacitor, which is the most complex one among the three kinds of electrical components, is specially chosen as an example to be printed on the paper. Figure 1A indicates the detailed dimensions of four designed capacitors with both in-plane and conventional sandwich patterns. Figure 1B illustrates the main preparation procedure for directly writing electrical components on paper by using GaIn_10_-based liquid metal ink. The process mainly falls into five steps: First, the capacitors including both in-plane and conventional sandwich patterns have been designed. For in-plane components, three different geometries are adopted. Second, the designed patterns of the capacitors are carved on the thin polyimide sheet (thickness of 0.1 mm) to prepare the mask which is used to accurately control the shape and size of the designed patterns on substrates. Third, a piece of A4 typing paper is chosen as the substrate. As is well known, the paper is one of the water absorbing materials. Hence for avoiding potential effect of the paper substrate on the conductive properties of the GaIn_10_-based liquid metal ink, a thin film of transparent silicone rubber serving as insulating layer was painted on the paper as shown in Figure 1. Fourth, the capacitors are directly written on paper after the silicone rubber becomes dry. Finally, a polyvinyl chloride (PVC) film should be covered on the liquid metal electrical components. On the one hand, the GaIn_10_-based liquid metal ink with a melting point of 289 K measured by the differential scanning calorimeter remains at a semi-liquid state around room temperature and therefore may not solidify after writing on a substrate unless it was subjected to an appropriate cooling before use. Hence, the metal sometimes may smear out due to mechanical stress. As a remedy, a PVC thin film can be covered on the wet patterns, which would protect the electrical components effectively. On the other hand, the Gallium oxide layer formed on the surface can be detached from the users, which further ensure the operational security of the gallium-based liquid metal ink. It is emphasized that a current lead should be used for the electrical characterization. Here, one side of the current lead is connected with the liquid metal based terminal of the components and covered by a PVC film together, and the other side is used to connect the probe or jig of the test devices. The thickness of the liquid metal ink layer, insulating varnish layer and PVC layer is about 50 µm, 100 µm and 100 µm, respectively. Figure 1C shows the exploded view of schematic illustration of an array of concentric circular patterns for electrical capacitors directly written on paper, which is prepared by the above preparation process. The paper-based flexible circuits mainly comprise four layers: paper substrate, insulating layer, electrical components, and covering film.

**Figure 2:**
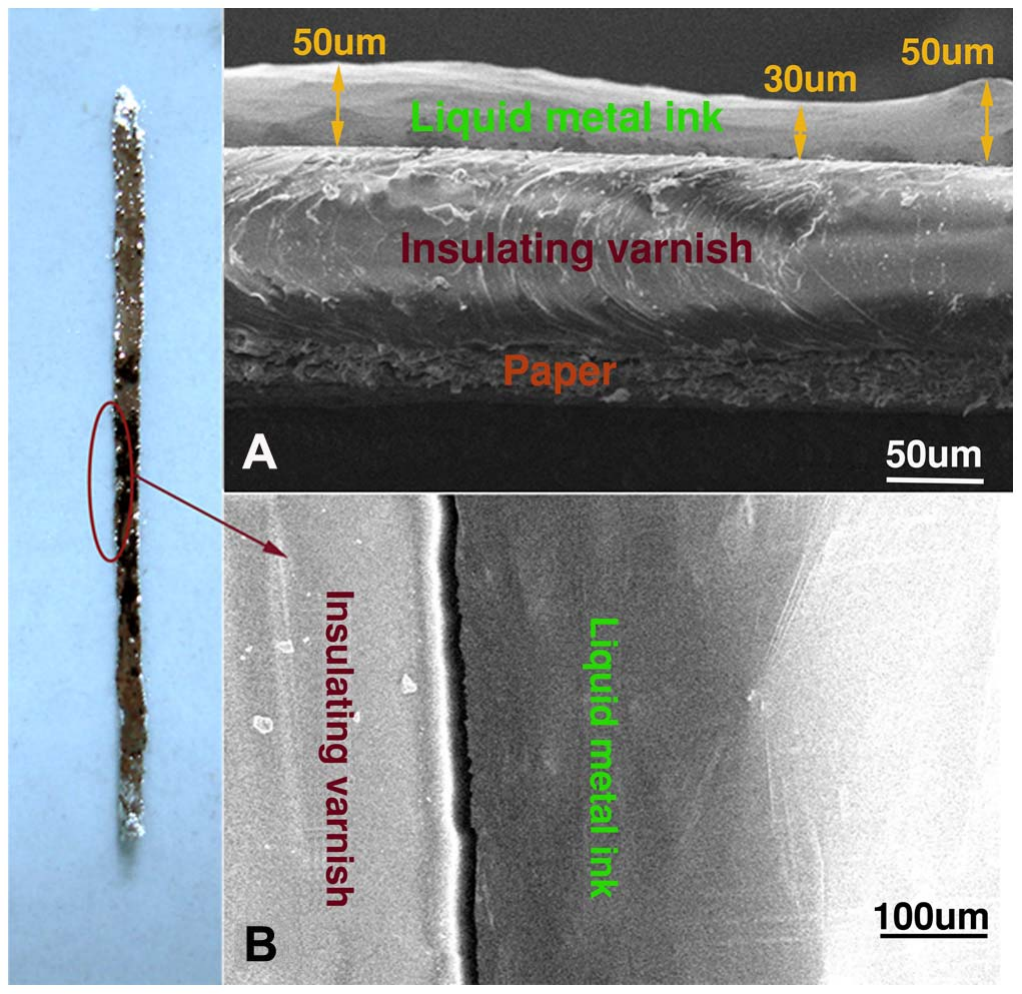
SEM graphs of section and surface topography of conductive line. (A) SEM graphs of section and (B) surface topography of conductive line written on paper by using GaIn_10_-based liquid metal ink. The thickness fluctuation range of conductive line is from 30 um to 50 um and the thickness of insulating varnish is about 100 um.

Figure 2 shows the optical image of a conductive line prepared by following the above procedure. This conductive line with width of about 2 mm is directly written on the typing paper by brush pen, with SEM graphs showing its cross section and surface topography in Figure 2A and Figure 2B, respectively. It can be observed that the average thickness of the conductive line written on the paper and the silicone rubber painted on the paper can reach about 40 µm and 100 µm, respectively. The silicone rubber painted on paper closely integrates with the liquid metal ink, which well proves again the excellent wettability of the metal ink with the silicone rubber painted on the paper.

### Direct Writing of Complex Circuits on Different Substrate Materials

**Figure 3:**
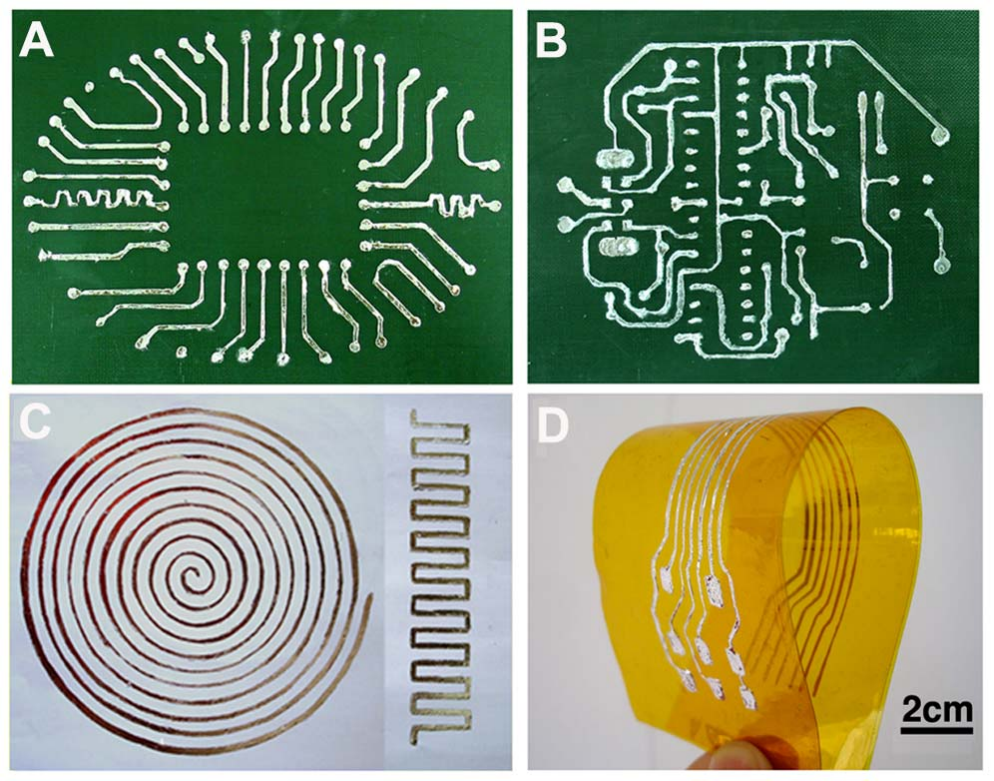
The printed circuits prepared by directly writing on different substrate materials with either soft or rigid properties which are expected to be used in future electronics. (A) – (B) Glass reinforced plastics (GRP). (C) The inductors directly written on paper. (D) Polyimide.

The perfect wettability of GaIn_10_-based liquid metal ink makes it possible to be used to directly and easily draw electrodes, electronic components and complex electrical circuits with various desired circuit lines and patterns on a desired substrate without any leakage, skipping or flowing. Figure 3 shows the printed electrodes, electrical components and complex electrical circuits prepared by directly writing on different substrate materials with either soft or rigid properties, which are widely used in electronics. Figure 3A and Figure 3B are the complex printed electrical circuits with a function of a Hi-Tech vector circuit and a crystal tester circuit, respectively, which are all directly written on glass reinforced plastics (GRP), while Figure 3C shows two kinds of inductors with different geometries made by the same preparation process (as shown in Figure 1). In addition, general electrical connecting lines are directly written on polyimide as shown in Figure 3D. Compared with the non-wetting pure GaIn_10_ alloys and low electrical conductive organic materials, the GaIn_10_-based liquid metal ink consisting of more than 99% GaIn_10_ alloy not only has a good wettability, but also owns low electrical resistivity. It is convinced that the above direct writing process can be used to effectively develop a wide variety of custom flexible or rigid electronic circuits in the near future. In this way, one can quickly turn around any design in short times from layout into direct writing prototypes. GaIn_10_-based liquid metal serving as a special metal ink for fabricating effective circuit board has rather important practical significance and broad application prospect.

### Wettability of GaIn_10_ Ink with Different Papers as Substrates

**Figure 4:**
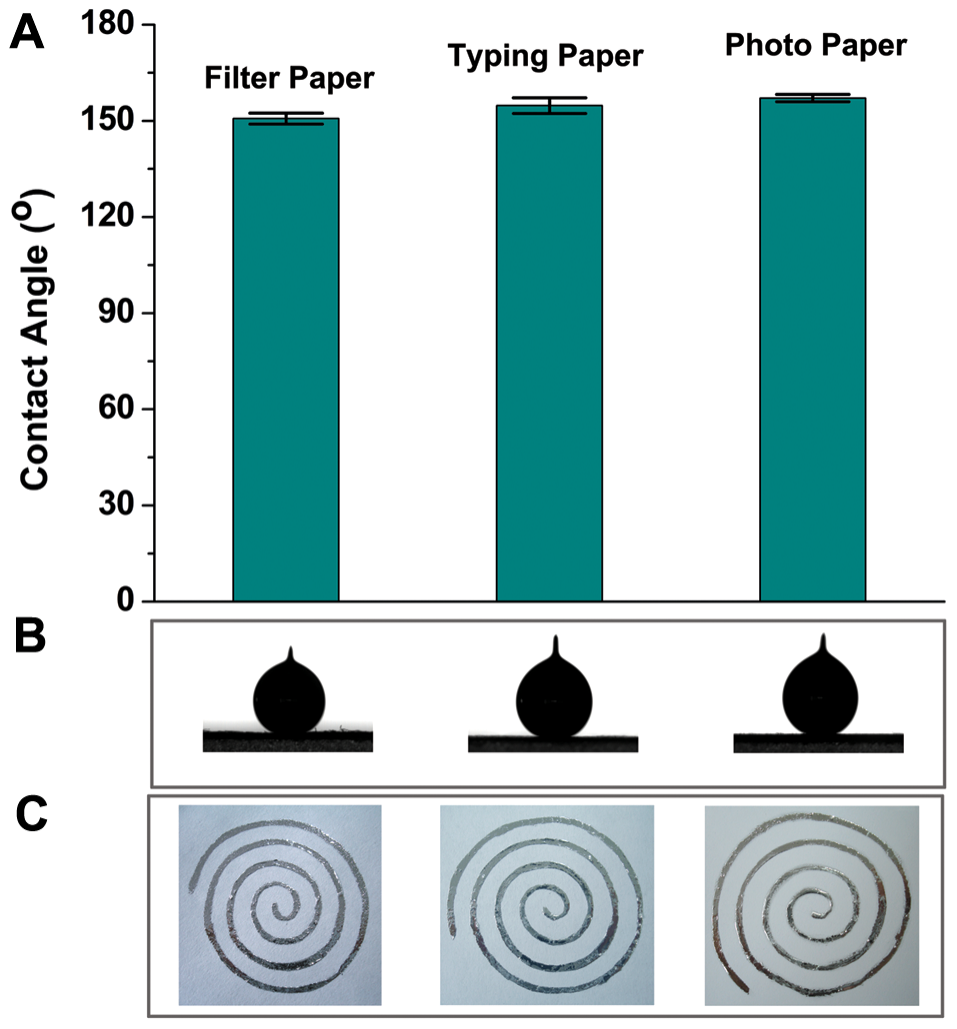
Wettability of GaIn_10_ ink with three different papers. (A) Contact angles variation and (B) droplets of ink with different papers. (C) The coils directly written on three different papers.

It is well-known that paper substrates offer tremendous advantages for fabricating printed electronic devices. Here three different papers including filter paper, typing paper and photo paper are employed to study their influences on the wettability of GaIn_10_ ink as shown in Figure 4. It can be seen that there is only a small change of contact angle on different papers as shown in Figure 4A and Figure 4B. The liquid solid interface all has a high contact angle which is helpful to obtain a narrow drawing track on paper. Figure 4C shows the inductors written on three different papers by using liquid metal ink, which indicates that GaIn_10_ ink can be easily written on three papers although there is a slight variation on contact angles.

### Stability Measurements of Electronic Components Directly Written on Paper

**Figure 5:**
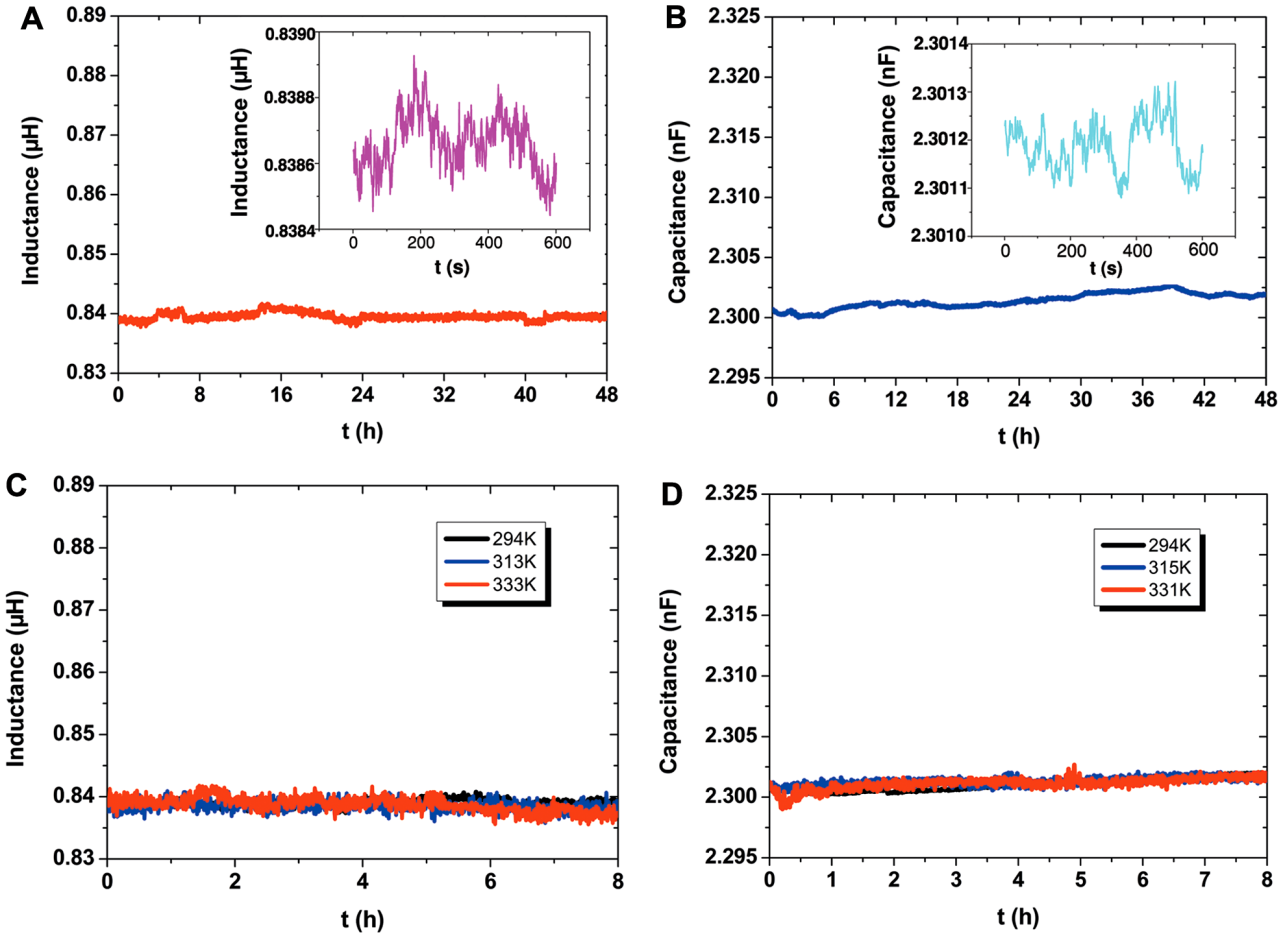
Stability measurements of electrical components by using a digital electric bridge. (A) The inductance with a coil-type structure and (B) the capacitance with a sandwich configuration are all directly written on paper by using liquid metal ink. (C) – (D) The capacitance and inductance of the electrical components as a function of temperature, respectively.

Considering the requirements from practical applications, it is necessary to comprehensively know more about the electronic components. The inductor adopts the coil-type structure as shown in Figure 3C, which includes 11 coils and its diameters start at 1 cm and increase by 1 cm. The line width is about 1 mm with an inaccuracy of about 10% which changes the line spacing and has little effect on the electrical measurement. The capacitor with a sandwich-like configuration as shown in Figure 1 uses the polyethylene thin film as dielectric layer which is about 10 µm thick, and the effective area is about 3 cm×3 cm. Figure 5A and Figure 5B present the value of inductance and capacitance as a function of working time with a data acquisition rate of 60 s when the electrical components keep on working for 48 h, and the inserts indicate the small variation of inductance and capacitance with a data acquisition rate of 1 s when the electrical components continuously work for 10 min. It is measured that the inductance and capacitance are about 0.8386 µH and 2.3012 nF as measured by LCR digital electric bridge with the measurement frequency and the level of 100 kHz and 0.3 V, respectively. The inductance and capacitance of the printed components have an inaccuracy of about 3% and 1% because of the impact of inaccuracy in directly written line dimensions, respectively. The inductance and capacitance of the printed components are all very stable with the working time even though they all show a small fluctuation as indicated by the insert. The accuracy of inductor and capacitor are about 0.043% and 0.25%, which is in accordance with the standard deviation. In addition, it is necessary to clarify the thermal stability of the ink because of its low melting point. Figure 5C and Figure 5D show the transient capacitance and inductance of the electrical components as a function of temperature, respectively. Here the maximum temperature is chosen as 60°C, which is the upper safe temperature applied to the printed components restricted by the paper substrates. It can be seen that the values of capacitance and inductance are all very stable with the temperature even though the electrical components keep on working for 8 h at each temperature. The low melting point of the ink does not have an impact on its thermal stability because the thickness of the electrical components is in micron and there is no fluidity of the ink, the structure cannot be changed easily when the working temperature is elevated. Moreover, the PVC film is covered to protect the electrical components. Hence, the electrical components including capacitor and inductor directly written on paper own a good stability and potential widespread adaptability, especially when used in some high frequency circuits.

### Parameter Justification of the Fabricated Electronic Elements

From the basic concept offered by the present method, it is feasible to directly write out various kinds of resistors, capacitors and inductors on paper with desired magnitudes by liquid metal ink. Table 1 summarizes the basic physics regarding the three electrical components. At the present stage, the thinnest lines directly written on paper by using GaIn_10_-based liquid metal ink can only reach 0.5 mm in width and 10 µm in thickness. In the near future, a pen with micro or even nano sized outlet can be fabricated to write the structure in pretty fine scale. Except for the geometric factors, the electrical resistivity can also be effectively changed by controlling the chemical components of the liquid metal ink. For example, a measured amount of silver or copper nano-particles can be added into liquid metal ink to prepare new liquid metal ink with a lower electrical resistivity. Some semiconductor nanoparticles can also be loaded to the liquid metal to generate new ink which has a higher resistivity or the semiconductor characteristics.

**Table 1:** Essential physical properties of three basic electronic components.

Electronic components	Units	Formula	Remarks
Resistor (*R*)	Ohm (Ω)	*R = ρLS^−1^*	*ρ*: Electrical resistivity (Ω·m)
			*L*: Length (m)
			*S*: Area (m^2^)
Capacitor (*C*)	Farad (F)	*C = ε_0_ε_r_ Sd^−1^*	*ε_0_*: 8.86×10^−12^ (Fm^−1^)
			*ε_r_*: Dielectric constant
			*S*: Effective area (m^2^)
			*d*: Thickness (m)
Inductor (*L*)	Henry (H)	*L = 0.8r^2^N^2^(6r+9l+10d)^−1^*	*r*: Average radius (m)
			*N*: Coil number
			*l*: Coil length (m)
			*d*: Coil thickness (m)

### Direct Writing of a Typical Functional Oscillating Circuit on Paper

**Figure 6:**
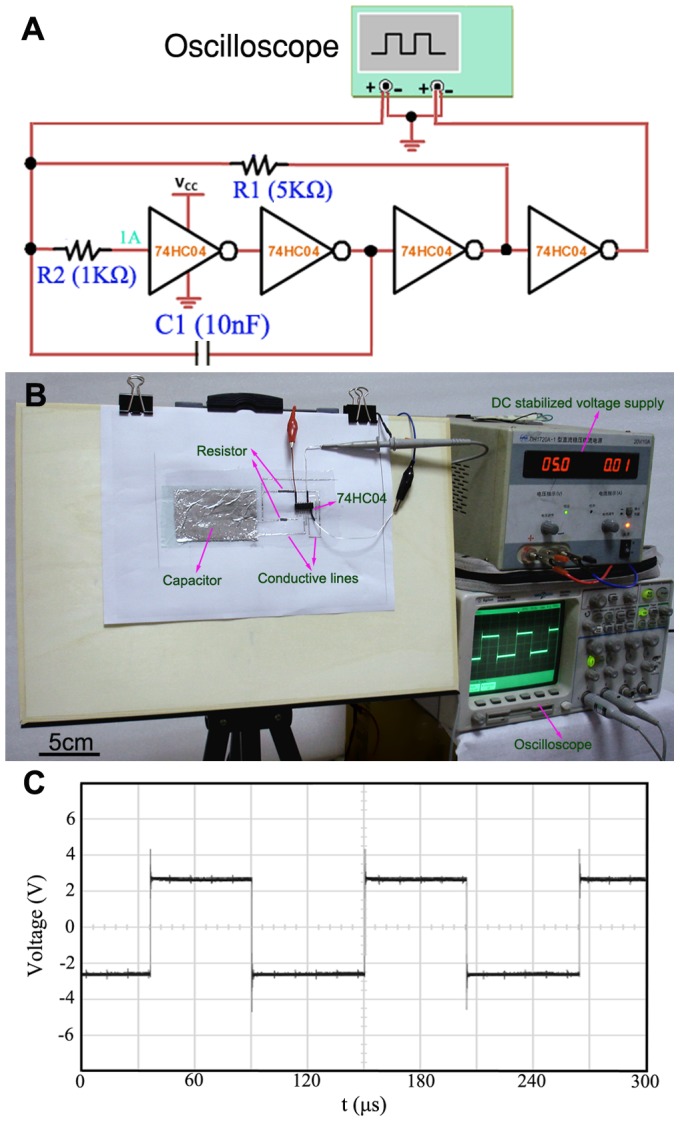
The typical LC oscillating circuit directly written on paper. (A) An electric schematic diagram of the typical LC oscillating circuit. (B) Palette like experimental platform for direct writing of a typical oscillating circuit. Here the two resistances and a capacitance included within the diagram are written directly on paper with electrically conductive silicone and gallium-based liquid metal ink, respectively. (C) The oscilloscope exhibits the result of a square wave with a frequency of 8.8 KHz. Here, an integrated chip of 74HC04 is driven by a supply voltage of 5 V.

To demonstrate the electronic device fabrication capabilities offered by the present writing approach, in the following, we designed a typical functional oscillating circuit including a capacitor of 10 nF and two resistors of 5 KΩ and 1 KΩ, respectively and an integrated chip of 74HC04 with a supply voltage of 5 V as shown in Figure 6A, which presents an electric schematic diagram of the typical oscillating circuit. Here the two resistances and a capacitance included within the diagram were written directly on paper with conductive silicone and GaIn_10_-based liquid metal ink. As is now understood by our measurement, the GaIn_10_-based liquid metal ink consisting of more than 99% GaIn_10_ alloy has a better electrical properties, which shows an electrical resistivity of about 34.5 µΩ·cm at 297 K measured by using four point probe method. Hence a resistance directly written by using GaIn_10_-based liquid metal ink is very small and cannot fulfill the request for practical purpose. As an alternative, the commercially available conductive silicone with an electrical resistivity of 0.02Ω·cm at room temperature was chosen to write a large resistor on paper. Figure 6B shows the photograph of the experimental platform which mainly includes an oscillating circuit directly written on paper, a DC stabilized power supply and an oscilloscope. For this oscillating circuit, the square wave with an oscillation frequency of 8.8 KHz can be observed by oscilloscope as shown in Figure 6C, which is in well agreement with the value of 9.1 KHz simulated by Multisim circuit-simulator software. It is observed that in this circuit all the electrical components including resistor, capacitor and conductor are directly written on the paper except an integrated chips (74HC04). The whole process looks just like making an oil painting. What is more important is that the oscillating circuit can be steadily running without additional signal interference.

## Discussion

**Figure 7:**
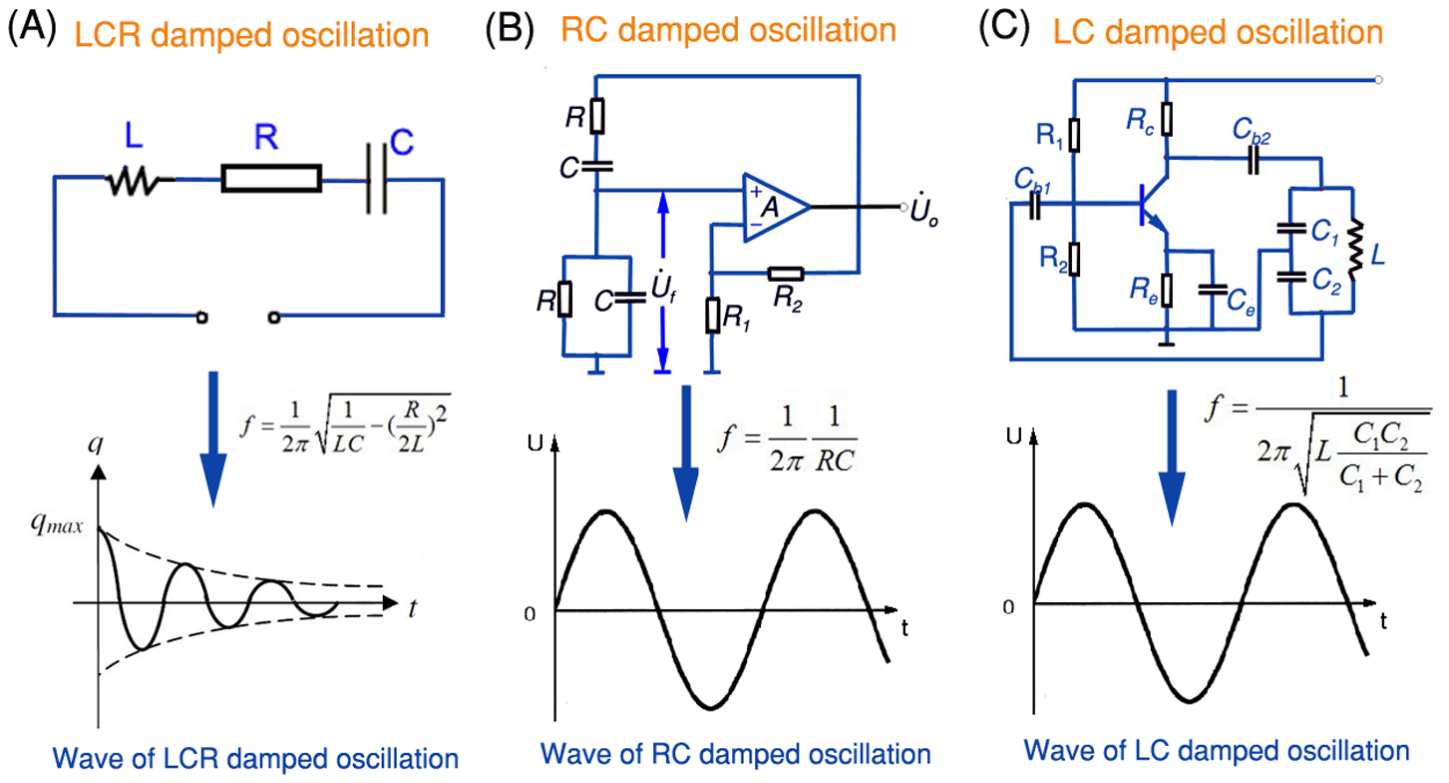
The Typical oscillating circuits and corresponding oscillating wave. (A) LCR damped oscillation. (B) RC damped oscillation. (A) LC damped oscillation.

Clearly, the present method paves the way to directly write out the most fundamental electrical elements and thus compose complex circuits on different substrate materials with a variety of desired lines and patterns. Along this way, much more functional electrical circuits can be directly written on paper. Figure 7 presents additional typical oscillating circuits which comprise three basic electrical components as illustrated above. The complicated oscillation circuits with different frequency and wave can be written on paper by using GaIn_10_-based liquid metal ink, conductive silicone ink and some other electrical devices such as integrated chips which have been commercialized and widely used in diverse fields. Thus it can be seen that the new method and basic principle have generalized purposes and can be extended to more industrial and business areas, even daily life. Such writing task can also be delivered to the computer in case of the user is not a good writer or drawer. A pre-designed computer program will drive the printer filled with desired liquid metal ink to directly print out the circuits. This may incubate the “Do it yourself” consumer electronics in the future.

In addition, as disclosed by the above fundamental experiments, many functional electrical devices can be made following the present basic concepts. GaIn_10_-based liquid metal ink combined with the conventional organic/inorganic ink, such as conducting polymer based composites [23], transparent conducting oxides (TCO) ink [34], P-type metal-oxide semiconductor (PMOS) ink, N-type metal-oxide semiconductor (NMOS) ink [35], [36], single-walled carbon nano-tubes [37], [38] and silicon or germanium semiconductor ink [39], can be used to directly write further more functional devices on paper or on some other substrate materials with either soft or rigid properties.

In the present work, the conductive lines and patterns are all directly written on paper by a brush pen and a mask. Based on the mask, the shape and the dimensions of the designed lines and patterns can be precisely written on substrates via the brush pen. However, there is a certain inaccuracy of the width and thickness of the lines directly written by hand writing. The reason lies in that the size of the pen-point, wettability and viscosity of the GaIn_10_ ink, the thickness of the mask, and the pressure as applied on the substrate are all the main factors affecting the resolution, edge roughness and line thickness of the conductive patterns. In order to fulfill the needs of further accuracy and micro/nano devices, several printing modalities, such as screen printing, 3D printing, laser printing, inkjet printing etc., should also be studied. Especially, inkjet printing, a contactless technique of printing which is widely used for desktop publishing, is worth of particular pursuing. Hence, various factors of the liquid metal ink, such as wettability, viscosity, conductivity etc., should be investigated and adjusted to satisfy the running of the inkjet printer. Clearly, tremendous efforts are urgently needed along this direction in the coming time.

## Conclusions

The present article demonstrated the low-cost and direct writing of fundamental electronic elements such as resistor, inductor and capacitor to compose functional circuits through the low melting point alloy ink. Such material remaining at a semi-liquid state around room temperature can be directly written on various either soft or rigid materials owing to its well controllable wettability. The conceptual experiments illustrated the good stability and potential widespread adaptability of the fabricated electronic components especially when used in some high frequency circuits. Hence, it is worth to mention that a landmark area of directly integrating functional circuits on paper or more substrates using room temperature liquid metal and its matching inks is in incubating.
